# A cytotoxic peptide-drug conjugate for tumor-specific delivery of co-injected molecules

**DOI:** 10.1371/journal.pone.0331564

**Published:** 2025-09-02

**Authors:** Norio Miyamura, Chisato M. Yamazaki, Yasuaki Anami, Kyoji Tsuchikama, Kazuki N. Sugahara

**Affiliations:** 1 Department of Surgery, Columbia University Vagelos College of Physicians and Surgeons, New York, New York, United States of America; 2 Texas Therapeutics Institute, The Brown Foundation Institute of Molecular Medicine, McGovern Medical School, The University of Texas Health Center at Houston, Houston, Texas, United States of America; Faculty of Medicine of Tunis, TUNISIA

## Abstract

An ideal cancer therapy enhances anti-tumor effects while minimizing side effects. iRGD, a non-cytotoxic peptide that activates a tumor-specific molecular transport machinery, promotes the penetration of co-injected drugs into tumor tissues. Clinical trials have demonstrated its potential as a tumor-specific delivery scaffold and potentiator of anti-cancer agents. In this study, we synthesized an iRGD conjugate containing monomethyl auristatin F (MMAF), a highly toxic antimitotic agent, and characterized its dual function as a tumor-specific cytotoxic agent and co-injected drug delivery scaffold. The iRGD-MMAF conjugate internalized and killed cultured tumor cells in an αv integrin-dependent manner. When injected systemically, iRGD-MMAF homed selectively to tumors in mice, and extensively spread in the extravascular tumor tissue in line with the tumor-penetrating capacity of iRGD. iRGD-MMAF also significantly enhanced tumor-specific entry of a co-injected molecule by serving as an effective drug delivery scaffold. The results indicate that a chemically modified iRGD peptide with an added therapeutic benefit retains its ability to deliver co-injected agents to tumors.

## Introduction

Cancer therapy remains challenged by suboptimal anti-cancer effects and significant off-target toxicity. One major cause is poor drug penetration into tumors. Solid tumors often have high interstitial fluid pressure, preventing systemic drugs from extravasating and spreading within the tumor tissue [1,2]. As a result, only 1–3% of the injected dose enters the tumor leaving a major proportion of the dose available for off-target toxicity [3]. These challenges are particularly exacerbated in desmoplastic tumors such as pancreatic ductal adenocarcinoma (PDAC).

The iRGD peptide greatly improves drug penetration into tumors providing a solution to the above issues. iRGD was originally identified as a group of peptides that greatly internalize into cancer cells (amino acid sequences: CRGDK/RGPD/EC) [4]. The internalization is mediated by a 3-step mechanism that involves 1) an RGD motif that binds to cell surface αv integrins, 2) proteases that cleave iRGD to activate a neuropilin-1 (NRP-1)-binding CRGDK/R fragment, and 3) the CRGDK/R fragment that triggers the formation of endosomal vesicles in an NRP-1-dependent manner. Some of the vesicles fuse with each other to form a cell penetration machinery that actively transports iRGD (and molecules linked to iRGD) through the cells [5,6]. When injected systemically, the RGD motif directs the peptide to αv integrins expressed on tumor blood vessels facilitating tumor-specific homing. Once the peptide is recruited to the tumor, it is proteolytically transformed into the CRGDK/R fragment to induce the penetration pathway for effective extravasation. The penetration cascade continues within the extravascular tumor tissue mediated by cells that express αv integrins and NRP-1, such as cancer-associated fibroblasts (CAFs) and cancer epithelial cells [7]. As such, iRGD greatly improves tumor-specific penetration of drugs that are directly linked to iRGD leading to enhanced anti-tumor effects without significantly increasing off-target toxicity [4,8].

The NRP-1-dependent penetration machinery transports bystander molecules in addition to iRGD. The vesicles that compose the machinery resemble macropinosomes, which engulf the extracellular fluid [6]. Thus, bystander molecules in the microenvironment are incorporated in the vesicles and co-transported with iRGD. This bystander effect allows iRGD to achieve tumor-specific tissue-penetrating delivery of co-injected free compounds [9]. iRGD enhances tumor entry of co-injected drugs by a few folds (or even more in certain cases) and potentiates the anti-tumor effects accordingly. In a phase 1b clinical trial performed in stage 4 PDAC patients, iRGD in combination with gemcitabine and nab-paclitaxel achieved a response rate of 59% and disease control rate of 90% [10], which are approximately twice as high as the rates reported in a historical landmark phase 3 trial that studied gemcitabine + nab-paclitaxel alone in the same patient population [11]. The addition of iRGD did not increase adverse events. The trial has now progressed to randomized double-blinded phase 2b studies (e.g., NCT05042128 ASCEND trial), which are reportedly showing promise based on an interim futility analysis.

While iRGD effectively delivers drugs to tumors, the peptide alone does not have meaningful toxicity against cancer cells [4,9,12]. The lack of cytotoxicity is beneficial to achieving safe drug delivery. On the contrary, an iRGD variant that is cytotoxic by nature may prove to be a more powerful drug delivery scaffold for cancer therapy. Here, we investigated the anti-cancer effect of an iRGD-drug conjugate and whether it delivers a co-injected molecule selectively to tumors.

## Materials and methods

### Synthesis of iRGD-conjugates

All information regarding synthesis procedures and characterization data for these compounds are provided in the Supporting Information.

### Tumor cells

M21L4 and M21L human melanoma cells, KRAS-Ink mouse PDAC cells, and AA0779−1 mouse CAFs were cultured in Dulbecco’s modified Eagle medium with 10% fetal bovine serum and a penicillin-streptomycin mixture. M21L4 and M21L cells were established as previously described [13]. KRAS-Ink cells were derived from PDAC tissue of *p48-CRE, LSL- Kras*^*G12D*^*, INK4a*^*flox*^ mice [14]. AA0779−1 cells were established from mice implanted with fresh primary human PDAC tissue and immortalized using a lentiviral transduction system of hTERT purchased from Applied Biological Materials (Richmond, BC) [7]. The cells tested negative for mycoplasma contamination. The cells were authenticated by ATCC (Manassas, VA).

### *In vitro* internalization assays

Fifty thousand cells were seeded on collagen I-coated coverslips (Neuvitro Corporation, Camas, WA). After 24 hr of incubation, the cells were treated with 10 μM FAM-MMAF or FAM-iRGD-MMAF at the indicated concentration for 3 hr. In some cases, 20 μg/ml of anti-αv integrin antibody (Ab) (ab63490 Abcam, Waltham, MA) or control IgG (PI31903, Thermo Fisher Scientific, Waltham, MA), or an equal amount of PBS was added to the media 2 hr prior to adding the drugs. The cells were fixed with 4% paraformaldehyde (PFA) for 15 min, washed in PBS three times, and stained with 4’,6-diamidino-2-phenylindole (DAPI; Thermo Fisher Scientific, Waltham, MA). In some experiments, the cell membrane was stained with CellMask Plasma Membrane Stains Deep Red (Thermo Fisher Scientific, Waltham, MA) prior to fixing the cells. The cells were imaged under an LSM 710 confocal microscope (Zeiss, Oberkochen, Germany). A ZEN 3.0 SR black edition software (Zeiss) was used for acquisition and analysis of the images.

### Cytotoxicity assay

Fifty thousand cells per well were cultured in 24-well plates for 24 hr. The cells were treated with 10 or 25 μM of FAM-MMAF or FAM-iRGD-MMAF for various durations. In some cases, an anti-αv integrin Ab (ab63490 Abcam), control IgG (PI31903), or PBS was added to the cultures 2 hr prior to adding the drugs. Cell survival was analyzed at the end of the study using Trypan Blue staining.

### Animal care and tumor mouse models

Tumor-bearing mouse models are widely recognized as a valuable system that closely mimics the human tumor microenvironment. We used tumor mice because complex *in vivo* kinetics such as tumor-specific homing, anti-tumor efficacy, and off-target side effects of drugs cannot be precisely studied without an *in vivo* model despite the recent advancement in *in vitro* systems. All animal experiments were performed according to procedures approved by the Institutional Animal Care and Use Committee (IACUC) at Columbia University (Protocol number: AC-AABU3706). All experiments are reported in accordance with Animal Research: Reporting of *In Vivo* Experiments (ARRIVE) guidelines. All efforts were made to minimize animal suffering and distress. Personnel involved in animal handling received comprehensive on-site training, including computer-based learning modules, hands-on training sessions, and ongoing education provided by veterinary and research staff. Animals were housed under a 12-hour light/dark cycle with unrestricted access to food and water. All the mice underwent a two-week acclimatization period in the animal holding room before the start of the experiments.

Mice bearing a subcutaneous PDAC tumor were prepared by injecting 5.0 x 10^5^ KRAS-Ink cells into the right flank of 8- to 10-week-old FVB/NJ mice. Mice were considered successfully engrafted when tumor formation was visibly confirmed, at which point, the mice were randomly numbered and assigned to a corresponding experimental group. The tumor mice were weighed twice daily for 2 days and then daily for 1-week post-surgery. From day 8, animals were weighed twice a week until the end of the experiment. Starting at 8 days post-surgery, tumor-size were monitored three times weekly. Tumors were not allowed to grow beyond 2 cm in diameter, and animals were monitored for signs of distress such as reduced body weight, cachexia, lack of mobility, reduced food and water intake or discomfort. Monitoring of tumor progression and health status of the mice were performed by investigators. The total duration of the experiment was within 19 days. The allocation of groups, drug administration, and measurements were conducted independently by at least two individuals to avoid bias. No mice were excluded from the analysis. The minimal sample size required to obtain a statistically meaningful result to answer our hypotheses was estimated based on similar studies performed in the past.

### *In vivo* drug homing assays

FAM-MMAF or FAM-iRGD-MMAF (4 μmol/kg) was injected into mice bearing a KRAS-Ink subcutaneous tumor via the tail vein. After 1 hr of circulation, the mice were perfused through the heart under deep anesthesia using PBS containing 1% bovine serum albumin (BSA), and tissues were collected. Each individual mouse was considered an experimental unit within the studies. The sample size was determined as discussed elsewhere. The tissues were fixed in 4% PFA overnight, washed with PBS three times, and maintained in 30% sucrose at 4°C until they sank. The tissues were then embedded in optimal cutting temperature compound to prepare 10 μm frozen sections. The sections were stained with anti-mouse CD31 Ab (MEC13.3, BD Pharmagen) followed by an appropriate secondary Ab tagged with Alexa Fluor 546 (Thermo Fisher Scientific) and DAPI. Images were taken with an LSM 710 confocal microscope system using a ZEN 3.0 SR black edition software.

### Tumor treatment studies

Mice bearing a KRAS-Ink subcutaneous tumor were randomized into three treatment arms, vehicle (PBS) alone, MMAF, and iRGD-MMAF. Mice received systemic therapy via tail vein every other day over 9 days at the dose indicated in the figures. The tumor size and body weight of the mice were measured in a time-dependent fashion. Each individual mouse was considered an experimental unit in the studies. The treatment studies were terminated according to the guidelines by the IACUC at Columbia University.

### *In vivo* permeability assay

Mice bearing a KRAS-Ink subcutaneous tumor were intravenously injected with 100 µl of 1% Evans blue (MP Biomedicals, Irvine, CA) dissolved in PBS followed 5 min later by 100 µl of PBS with or without MMAF or iRGD-MMAF at a dose of 4 μmol/kg via tail vein. After 40 min, the mice were perfused through the heart under deep anesthesia with PBS containing 1% BSA. Tissues were collected for macroscopic imaging and Evans blue quantification. Each individual mouse was considered an experimental unit within the studies. The sample size was determined as discussed elsewhere. The tissues were then submerged in 1 ml of N,Ndimethylformamide for 24 hr at 37˚C to extract Evans blue. The dye was quantified by measuring the absorbance at 600 nm with a spectrophotometer and normalizing the values to the tissue weight [9].

### Statistical analysis

One-way or 2-way analysis of variance (ANOVA) was used to compare three or more groups with a normal distribution. All statistics were performed using GraphPad Prism (Ver. 8.4.3).

## Results

### Design of iRGD-drug conjugates

**Fig 1:**
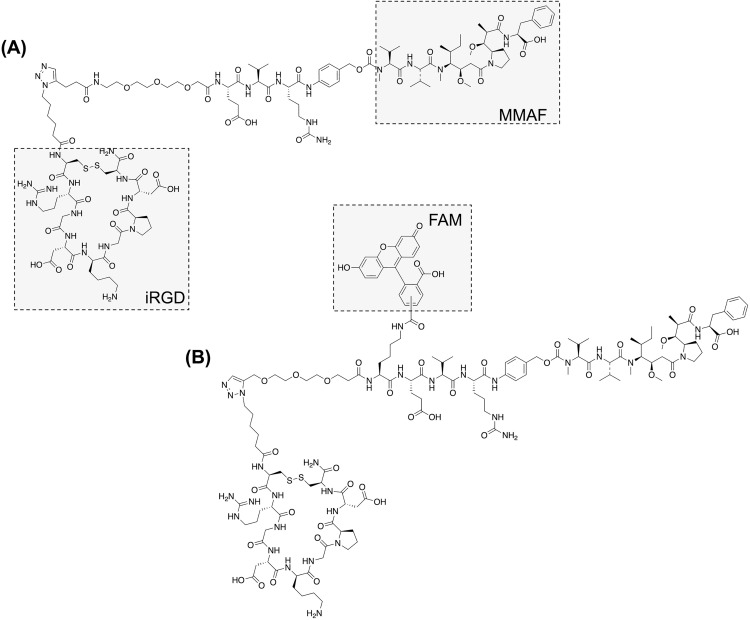
Chemical structure of iRGD-MMAF conjugates. (A) iRGD-MMAF. (B) FAM-iRGD-MMAF.

Since iRGD contains multiple reactive side chains that are essential for its function (e.g., lysine, aspartic acid), covalent linking of an anticancer drug to iRGD must avoid altering these critical moieties. To this end, we designed iRGD drug conjugates and synthetic schemes based on the orthogonal alkyne–azide click reaction, as illustrated in Fig 1 and SI. The conjugates consist of the tumor-penetrating iRGD peptide (cyclic -CRGDKGPDC-), a glutamic acid–valine–citrulline (EVCit) tripeptide linker [15–17], and monomethyl auristatin F (MMAF, Fig 1A and 1B). MMAF is a potent tubulin inhibitor commonly used as a payload of antibody–drug conjugates (ADCs), which requires precise, target-specific delivery due to its high toxicity. The EVC linker was chosen to enable stable systemic delivery and enhanced drug release in tumor tissue [16,17]. In one of the conjugates, we attached 5-carboxyfluorescein (5-FAM) to the side chain of a lysine residue introduced at the *N*-terminus of the Glu-Val-Cit sequence (Fig 1B). This fluorescently labeled iRGD–MMAF conjugate was used for in vitro and in vivo imaging, while the non-labeled iRGD–MMAF was used for cytotoxicity assays and in vivo treatment studies (See S1 Information for detailed synthesis procedures and characterization data for these compounds).

### αv integrin-dependent internalization and cytotoxicity of iRGD-MMAF

**Fig 2:**
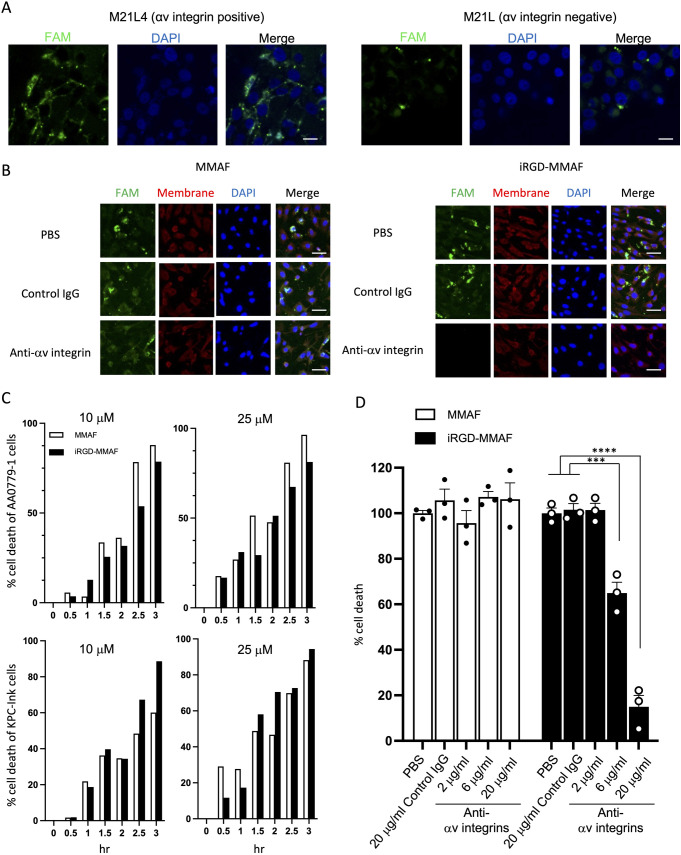
αv integrin-dependent internalization and cytotoxicity of iRGD-MMAF. (A) Confocal images of αv integrin-positive M21L4 and αv integrin-negative M21L human melanoma cells treated with FAM-labeled iRGD-MMAF (green). Scale bars, 50 μm. Blue, DAPI. Representative images from 3 biological replicates are shown. (B) Confocal images of KRAS-Ink mouse PDAC cells treated with FAM-labeled free MMAF or iRGD-MMAF in the presence or absence of an anti-αv integrin blocking Ab or a control IgG. Green, FAM-labeled drugs; red, cell membrane; blue, DAPI. Scale bars, 20 μm. Representative images from 3 biological replicates are shown. (C) Cytotoxicity of plain MMAF and iRGD-MMAF at 10 and 25 μM was assessed in AA0779−1 mouse CAFs and KRAS-Ink cells at different time points. (D) αv integrin-dependent cytotoxicity of MMAF or iRGD-MMAF. AA0779−1 cells were treated for 1.5 hr with 10 or 25 μM of MMAF or iRGD-MMAF in the presence or absence of an anti-αv integrin blocking Ab or a control IgG. Results from 3 biological replicates are shown. Error bars, mean ± standard error. Statistical analysis, 2-way ANOVA; ***p < 0.0005, ****p < 0.0001.

We studied whether the conjugate would internalize into cultured cancer cells in an αv integrin-dependent manner using variants of M21 human melanoma cells that either express high levels of αv integrins (M21L4) or lack the integrins on the cell surface (M21L) [4,13,18]. To visualize the internalization of iRGD-MMAF, we used the fluorescein-labelled iRGD-MMAF (FAM-iRGD-MMAF). The FAM-iRGD-MMAF effectively internalized into M21L4 cells, while it minimally entered M21L cells (Fig 2A). FAM-iRGD-MMAF also entered mouse KRAS-Ink PDAC cells derived from transgenic *p48-CRE, LSL-Kras*^*G12D*^*, INK4a*^*flox*^ mice (Fig 2B, right panels) [14]. The KRAS-Ink cells are known to express both αv integrins and NRP-1 [14]. The entry was inhibited by an anti-αv integrin blocking Ab. FAM-MMAF that lacked an iRGD peptide entered KRAS-Ink cells to some extent, but the entry was not inhibited by the anti-αv integrin Ab (Fig 2B, left panels). These results indicate that FAM-iRGD-MMAF internalized into cancer cells in an αv integrin-dependent manner.

iRGD-MMAF time and dose dependently killed KRAS-Ink PDAC cells and AA0779−1 mouse CAFs established from orthotopic tumors prepared in mice using 0779E patient-derived human PDAC cells (Fig 2C) [19]. There was no major difference in the potency of *in vitro* cytotoxicity between the iRGD-MMAF conjugate and plain MMAF. However, the cytotoxic effect of iRGD-MMAF was dose-dependently inhibited by an anti-αv integrin blocking Ab, while the effect of plain MMAF was not (Fig 2D). Collectively, the results show that iRGD-MMAF enters and kills cells in an αv integrin-dependent manner strongly suggesting that the conjugate retains iRGD functions.

### *In vivo* biodistribution and anti-tumor effects of iRGD-MMAF

**Fig 3:**
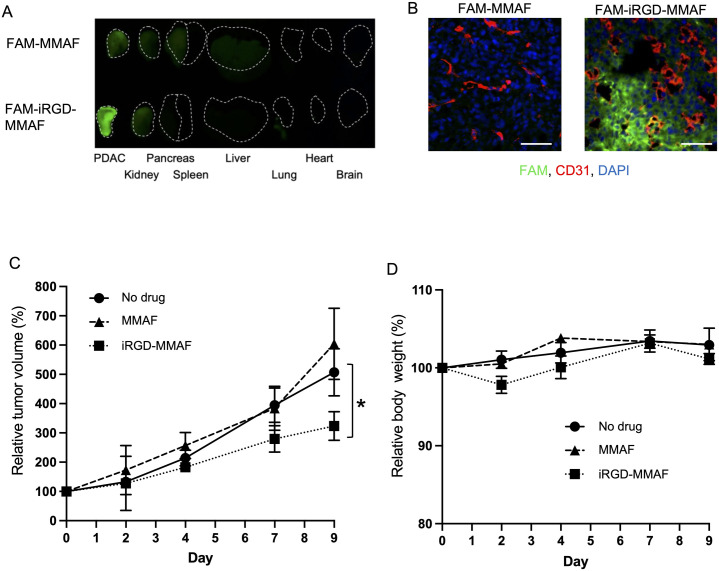
Tumor-specific homing and anti-tumor effects of iRGD-MMAF. (A) Fluorescence images showing tumor-specific homing of FAM-iRGD-MMAF. Mice bearing subcutaneous KRAS-Ink tumors were intravenously injected with 4 μmol/kg FAM-MMAF or FAM-iRGD-MMAF (green). Tissues were harvested 1 hr later and imaged under a fluorescence imager. Dash lines outline the tissues. Representative images from 3 biological replicates are shown. (B) Confocal images of the PDAC tumors shown in (A). Green, FAM-labeled drugs; red, CD31; blue, DAPI. Scale bars, 50 μm. (C) Tumor treatment study with plain MMAF and iRGD-MMAF. Mice bearing subcutaneous KRAS-Ink tumors were intravenously treated with 4 μmol/kg of MMAF or iRGD-MMAF every other day for 9 days. Control and iRGD-MMAF: n = 3, MMAF: n = 4. Error bars, mean ± standard error. Statistical analysis, one-way ANOVA; *p < 0.05. (D) Body weight changes of the mice treated in (C).

Intravenously injected FAM-iRGD-MMAF strongly accumulated into subcutaneous tumors in mice prepared with KRAS-Ink cells (Fig 3A). No obvious accumulation of FAM-iRGD-MMAF was noted in normal organs except for a minor signal in the kidney in line with renal clearance of small molecules [9]. In contrast, intravenously injected FAM-MMAF only weakly entered the tumors and provided some background in the liver. Immunofluorescence confirmed these results showing strong FAM signals in tumors from FAM-iRGD-MMAF-injected mice, but not those from FAM-MMAF-injected mice (Fig 3B). Of note, the FAM signals in the FAM-iRGD-MMAF group were found to be widely distributed in the extravascular space consistent with the tissue-penetrating capacity of the iRGD peptide. In line with these results, systemic treatment with iRGD-MMAF reduced the growth of KRAS-Ink tumors in mice (Fig 3C, S1 Table). In contrast, plain MMAF at the same dose had no effect on the tumor growth. iRGD monotherapy was not tested because it has been repeatedly shown in mice and humans to lack tumoricidal activities [e.g., 4,7,9,10,20,21]. Despite the enhanced anti-tumor effect, iRGD-MMAF did not cause any body weight loss in the mice in agreement with its tumor specificity.

### Tumor-specific delivery of a co-injected molecule with iRGD-MMAF

**Fig 4:**
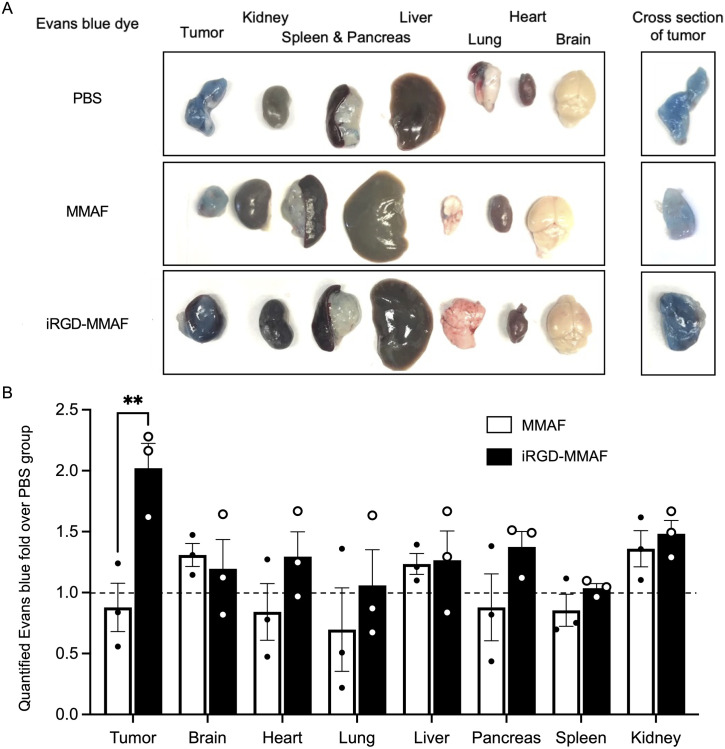
Tumor-specific delivery of a co-injected dye with iRGD-MMAF. Mice bearing subcutaneous KRAS-Ink tumors were intravenously injected with 100 μl of 1% Evans blue followed 5 min later by 4 μmol/kg of MMAF or iRGD-MMAF or an equivalent volume of PBS. Tissues were harvested 40 min later after cardiac perfusion with PBS containing 1% BSA. Representative results from 3 biological replicates are shown. (A) Images of the harvested tissues. The right-most panels show cross section images of the tumors that were sliced at 1.3 mm thickness. (B) Evans blue in the tissues was extracted and quantified based on absorbance at OD600. The values were normalized to tissue wet weight. Error bars, mean ± standard error. Statistical analysis, 2-way ANOVA; **p < 0.01.

The tissue-penetrating effect of iRGD-MMAF encouraged us to test whether the conjugate delivers co-injected agents selectively to tumors. We employed our modified Evans blue assay in which an albumin-binding dye (Evans blue) is used as a co-injected tracer of iRGD effects [9]. iRGD-MMAF caused significant accumulation of intravenously co-injected Evans blue into KRAS-Ink tumors in mice, while plain MMAF did not (Fig 4A). The conjugate enhanced dye entry into the tumors by approximately 2 folds compared to plain MMAF without causing significant changes in normal organs (Fig 4B). These results strongly suggest that iRGD-MMAF delivers co-injected drugs selectively to tumors.

## Discussion

The aim of this study was to characterize iRGD-MMAF and its capacity as a tumor-specific drug delivery scaffold. We chemically conjugated an iRGD peptide to a highly cytotoxic molecule MMAF using an EVC linker. The EVC linker facilitates effective in vivo drug delivery by providing stability in the circulation and high susceptibility to cathepsin B within tumors [17]. The iRGD-MMAF conjugate showed αv integrin-dependent internalization and cytotoxicity in cultured cells indicating proper iRGD functions. The conjugate also homed selectively and widely into the tumor in PDAC mice and showed enhanced anti-tumor effects compared to plain MMAF without causing major off-target effects. The in vitro and in vivo cytotoxicity against tumor cells was derived from MMAF and not iRGD because iRGD itself does not have anti-tumor effects [4,7,9,10,20,21] unlike some of the RGD-mimetics [22,23]. These results strongly suggest that the conjugate used iRGD to effectively and stably reach the tumor, and released cytotoxic MMAF within the tumor cells.

The αv integrin-dependent activities of iRGD-MMAF indicate that iRGD properly retained its cyclic structure in the conjugate. RGD peptides, in their linear form, have low affinity to αv integrins when a lysine residue succeeds the RGD motif [24]. iRGD is no exception [4]. However, when iRGD is cyclized, it acquires a peculiar horseshoe shape that allows the RGD motif to fit into the cleft of αv integrins with a nanomolar binding affinity [25]. The horseshoe shape is also critical for subsequent NRP-1-mediated activities because the conformation exposes the lysine-glycine bond outside of the integrin cleft to facilitate proteolytic cleavage that releases the NRP-1-binding CRGDK fragment [25]. While inhibitors to confirm NRP-1 dependency were not used in the current study, the NRP-1-mediated tissue-penetrating pathway was clearly activated by iRGD-MMAF because it extensively spread in the extravascular tumor tissue and doubled the amount of co-injected dye entry into tumors, effects that are not achieved by traditional RGD peptides that lack an NRP-1-binding motif [9].

Co-injecting iRGD is far more versatile and straightforward than conjugating iRGD to a drug to achieve tumor-specific drug delivery. Chemical modification can change the nature of the drug as well as iRGD, which can lead to diminished efficacy or unsuccessful drug delivery. Each chemical modification leads to a new drug entity, which can delay clinical translation. However, conjugation-based drug delivery may be beneficial in some cases. The use of a highly toxic compound, such as MMAF we used in this study, is one example. Even if iRGD enhances tumor entry of a co-injected drug, a major proportion of the drug will still remain in the circulation to cause off-target toxicity making it risky to co-inject an extremely toxic agent. Indeed, both in mice and in patients, co-injection of iRGD did not increase off-target toxicity but did not reduce it either, suggesting that side effects will likely limit the use of highly toxic drugs in the co-injection format. Conjugating iRGD to a drug to mask its toxicity until it is delivered to the target may allow the use of highly toxic agents that may not be compatible with iRGD co-injection.

While iRGD-MMAF showed promise as a tumor-specific cytotoxic agent in this study, further characterization is required for clinical translation. Examples of pending analyses include immunogenicity and pharmacokinetics (PK). In particular, PK studies are critical for optimizing the dosing schedule. Here, we intravenously injected the conjugate every other day at a dose of 4 μmol/kg following previous iRGD protocols [4,7,9]. In mice, the average plasma half-life of plain iRGD is 20–25 min at this dose [26]. However, the tumor-penetration effect remains for up to 24 h, indicating that circulating cargos can still be transported into the tumor within this time frame despite the short half-life of the peptide. The effect can be longer in larger animals, as the half-life of plain iRGD was about 30 min in rats, 40 min in dogs, and 96–108 min in humans [26]. To fully utilize these iRGD properties for conjugated MMAF delivery, chemically extending the half-life of iRGD-MMAF may be necessary as we expect the current formulation to have a short half-life given its relatively low molecular weight of 2540.98 (plain iRGD: 989.1). Previous work suggests various approaches in this regard such as adding a PEG, facilitating piggyback on albumin with an extra-cysteine, and using nanocarriers [4,27]. Optimizing the PK for effective therapy is a key for translation as frequent injections to compensate for a short half-life is not realistic in a clinical setting. An alternative is to utilize devices such as an infusion pump that delivers drugs over an extended time, which is in clinical use for small molecule drugs.

The ability of iRGD-MMAF to deliver a co-injected dye to tumors indicates that modification of iRGD, if properly performed, does not interfere with its bystander properties. This finding has important implications. The iRGD peptide that is currently in clinical trials is solely used as a co-injected drug delivery scaffold. The results shown in the current study suggests an option to incorporate additional functions to iRGD to provide an added benefit. Adding cytotoxicity as in this study, and biological effects, such as stromal modification and immunostimulation, are valid options. Adding imaging properties can facilitate simultaneous tumor-specific imaging and therapy to predict and validate therapeutic efficacy. While cargos and linkers for conjugation should be carefully selected not to interfere with iRGD properties, this study demonstrates that simultaneously delivering conjugated and co-injected drugs can be done and may expand the utility of the iRGD peptide as a drug delivery molecule. A multi-functional iRGD compound may further improve cancer management.

## Supporting information

S1 Information Information for detailed synthesis procedures and characterization data for compounds. (DOCX)

S1 Table Tumor size in mice with KRAS-Ink tumors following systemic treatment with MMAF or iRGD-MMAF. (DOCX)
