# Supplementary material for: A cytotoxic peptide-drug conjugate for tumor-specific delivery of co-injected molecules
Source: PLoS One. 2025 Sep 2;20(9):e0331564. doi: 10.1371/journal.pone.0331564 (PMC12404482; doi:10.1371/journal.pone.0331564)

**Information for detailed synthesis procedures and characterization data for compounds.**

**General information**

Unless otherwise noted, all materials for chemical synthesis were purchased from commercial suppliers (for example, Acros Organics, AnaSpec, Broadpharm, Chem-Impex International, Thermo Fisher Scientific, Levena Biopharma, Millipore Sigma, and TCI America) and used as received. All anhydrous solvents were purchased and stored over activated molecular sieves under argon atmosphere.

Analytical reverse-phase high performance liquid chromatography (RP-HPLC) was performed using an Agilent LC-MS system consisting of a 1100 HPLC and a 1946D single quadrupole electrospray ionization (ESI) mass spectrometer equipped with a C18 reverse-phase column (Accucore C18 column, 3×50 mm, 2.6 µm, Thermo Scientific). Standard analysis conditions for organic molecules were as follows: flow rate = 0.5 mL/min; solvent A = water containing 0.1% formic acid; solvent B = acetonitrile containing 0.1% formic acid. Compounds were analyzed using a linear gradient and monitored with UV detection at 210 and 254 nm. Preparative HPLC was performed using a Breeze HPLC system (Waters) equipped with a C18 reverse-phase column (SunFire Prep C18 OBD, 19×150 mm, 5.0 µm; Waters). Standard purification conditions were as follows: flow rate = 20 mL/min; solvent A = water containing 0.05% trifluoroacetic acid (TFA); solvent B = acetonitrile containing 0.05% TFA. Compounds were analyzed using a linear gradient and monitored with UV detection at 210 and 254 nm. In all cases, fractions were analyzed off-line using the LC-MS for purity confirmation and those containing a desired product were lyophilized using a Labconco Freezone 4.5 Liter Benchtop Freeze Dry System.

**Synthesis**

Synthesis of azido-iRGD (S3). Reagents and conditions: (a) TFA:TIPS (95:5), 4 hours, room temp; (b) iodine, 50% MeCN/water, room temp, <5 min.

**Synthesis of azidohexanoyl-Cys(Trt)-Arg(Pmc)-Gly-Asp(O*t*-Bu)-Lys(Boc)-Gly-Pro-Asp(O*t*-Bu)-Cys(Trt)-Rink amide AM resin (S1).**

Fmoc-Rink amide AM resin (0.2 mmol) was swollen by incubating in dichloromethane (DCM, 6 mL) for 1 h at room temperature. The solvent was drained, and the resin was washed with DCM (5×5 mL) and DMF (5×5 mL). To remove a Fmoc-protecting group at each coupling step, the resin was treated with piperidine (5 mL of 20% in DMF) for 20 min and washed thoroughly with DMF (5×5 mL). Fmoc-protected amino acid (4 equiv.) was pre-activated by being mixed with 1-[bis(dimethylamino)methylene]-1H-1,2,3-triazolo[4,5-b]pyridinium 3-oxid hexafluorophosphate (HATU, 4 equiv.) and *N*,*N*-diisopropylethylamine (DIPEA, 6 equiv.) in DMF for 2–5 min, and the cocktail was used for coupling (conditions: room temperature, 1 hour). The completion of the reaction was verified by the Kaiser test. After each coupling step, the coupling cocktail was drained, and the resin was washed with DMF (3×5 mL). After completing peptide elongation and subsequent removal of the Fmoc group, an azide group was introduced to the *N*-terminus of the peptide using 6-azidohexanoic acid under the same coupling conditions.

**Synthesis of azidohexanoyl-Cys-Arg-Gly-Asp-Lys-Gly-Pro-Asp-Cys (S2).**

**S1** was washed with DMF (3×5 mL) and DCM (3×5 mL) and dried in vacuo. For cleavage from the resin and deprotection of the protecting groups, a mixture of ice-cold trifluoroacetic acid (TFA, 95%, 4.75 mL) and triisopropylsilane (TIPS, 5%, 0.25 mL) was added to the dried resin and gently stirred for 4 hours at room temperature. The reaction mixture was filtered, and the crude peptide was precipitated with cold diethyl ether (35 mL) followed by centrifugation at 2,000 g for 5 minuets. The preceptee was washed by cold diethyl ether (35 mL) and centrifugation at 2,000 g for 5 minuets, three times. The crude peptide was dried in vacuo.

**Synthesis of azido-iRGD (S3).**

The dried crude peptide was dissolved in 100 mL of 50% acetonitrile (MeCN)/water. I_2_ (125 mg/mL in MeOH) was added dropwise to the solution until the solvent takes on a color. To quench the reaction, sodium ascorbate (50 mg/mL in water) was added. After the mixture was concentrated, the crude products were purified by preparative RP-HPLC to obtain analytically pure **S3** (58.2 mg, 27% overall yield, calculated based on the resin loading rate). Purity was confirmed by LC-MS. Off-white powder. MS (ESI) Calcd. For C_41_H_68_N_17_O_14_S_2_ [M+H]^+^: 1086.46. Found: 1086.4.

Synthesis of Alkyne-PEG3-Glu-Val-Cit-PABC-MMAF (S8): (a) 1% TFA/DCM, 1 hour, room temp; (b) *p*-aminobenzyl alcohol, EEDQ, DCM/MeOH(2:1), room temp, overnight; (c) bis(4-nitrophenyl) carbonate, DMAP, DMF, room temp, 2 hours; (d) MMAF, DIPEA, HOAt, DMF, 37 °C, 3 hours; (e) 50% TFA/DCM, room temp, 1 hour.

**Synthesis of Alkyne-PEG3-Glu(O*t*-Bu)-Val-Cit-OH (S4).**

2-Chlorotrityl chloride resin (1 g, 1.14 mmol, AnaSpec) and Fmoc-Cit-OH (678.8 mg, 1.71 mmol) were added to a manual solid-phase reactor containing DIPEA (793 µL, 4.56 mmol) and DMF (5 mL) and agitated for 2 hours. MeOH (300 µL) was added to the resin and agitated for 20 min. The solvent was drained, and the resin was washed with DMF (5×5 mL) and DCM (5×5 mL). Approximately half of the resin (0.57 mmol) was used to construct **S4**. To remove a Fmoc-protecting group after each coupling, the resin was treated with 20% piperidine/DMF (5 mL) for 20 min and washed with DMF (5×3 mL) and DCM (5×3 mL). 4-Pentynoic acid (3 equiv.), Fmoc-NH-PEG3-CH_2_COOH (Broad Pharm, 2 equiv.), Fmoc-Glu(O*t*-Bu)-OH (3 equiv.), or Fmoc-Val-OH (3 equiv.) was preactivated using HATU (3 equiv.) and DIPEA (4.5 equiv.) in DMF for 5 min, and the cocktail was added to the resin. The resin was agitated at room temperature for 1.5 hours. Completion of the coupling was verified by the Kaiser test. After each coupling step, the coupling cocktail was drained, and the resin was washed with DMF (5×3 mL) and DCM (5×3 mL). The resulting protected peptide resin was dried in vacuo and treated with a cocktail of 1% TFA/DCM at room temperature for 1 hour. The solution was concentrated in vacuo and the crude peptide was precipitated with cold diethyl ether (5–6 mL) followed by centrifugation at 2,000 × g for 3 minutes. The precepitate was washed by cold diethyl ether and centrifugation at 2,000 g for 5 minutes, three times. The resulting crude products were dried in vacuo and then used immediately in the next step without purification.

**Synthesis of Alkyne-PEG3-Glu(O*t*-Bu)-Val-Cit-PABC-OH (S5).**

To a solution of crude **S4** (218.6 mg, 0.3 mmol) in DCM/MeOH(2:1, 3.2 mL) were added *p*-aminobenzyl alcohol (110.8 mg, 0.9 mmol) and EEDQ (445.14 mg, 1.8 mmol). After being stirred in the dark at room temperature overnight, the solution was concentrated in vacuo and the crude peptide was precipitated with cold diethyl ether (20 mL) followed by centrifugation at 2,000 ×g for 3 min (10 times). The resulting crude peptide was dried in vacuo and then used immediately in the next step without purification.

**Synthesis of Alkyne-PEG3-Glu(O*t*-Bu)-Val-Cit-PABC-PNP (S6).**

Bis(2,4-dinitrophenyl) carbonate (471.5 mg, 1.55 mmol) and DMAP (75.7 mg, 0.62 mmol) were added to a solution of the crude peptide **S5** (256.2 mg, 0.31 mmol) in DMF (2.4 mL), and the mixture was stirred at room temperature for 2 hours under Ar. The reaction was quenched with 1% HCl/ACN (2.4 mL) at 0 ºC, then the crude products were purified by preparative RP-HPLC to afford analytically pure peptide **S6** (119.6 mg, 39% for the 2 steps). Purity was confirmed by LC-MS. White powder. MS (ESI) Calcd. For C_47_H_67_N_8_O_16_ [M+H]^+^: 999.47. Found: 999.3.

**Synthesis of Alkyne-PEG3-Glu(OtBu)-Val-Cit-PABC-MMAF (S7).**

A solution of **S6** (30 mg, 30 µmol) in DMF (300 µL) was mixed with monomethyl auristatin F TFA salt (34.5 mg, 40 µmol), HOAt (8.2 mg, 60 µmol), and DIPEA (26.1 µL, 150 µmol). The resulting mixture was stirred for 3 hours at 37 °C. The crude products were dried and DIPEA was removed in vacuo. The crude products were used for next step without purification.

**Synthesis of Alkyne-PEG3-Glu-Val-Cit-PABC-MMAF (S8).**

TFA (300 µL) and DCM (300 µL) were mixed and added to the dried crude compound **S7** at 0 ºC. After being stirred at room temperature for 1 h, the reaction mixture was concentrated in vacuo and the crude peptide was precipitated with cold diethyl ether (18­­–20 mL) followed by centrifugation at 2,000 × g for 3 min (3 times). The resulting crude products were dried in vacuo, and then was purified by preparative RP-HPLC to afford analytically pure peptide **S8** (26.3 mg, 57% for the 2 steps). Purity was confirmed by LC-MS. White powder. MS (ESI) Calcd. For C_76_H_119_N_12_O_21_ [M+H]^+^: 1535.86. Found: 1535.7.

Synthesis of iRGD-MMAF. Reagents and conditions: (a) phosphate buffer (pH 7), TBTA, CuSO_4_, ascorbic acid, DMSO, 37 ºC, argon atmosphere, 1 hour.

**Synthesis of iRGD-MMAF.**

The reaction mixture of **S8** (10 mM in DMSO, 0.8 mL), **S3** (10 mM in DMSO, 1.6 mL), 1 M phosphate buffer (pH 7, 2.4 mL), Tris((1-benzyl-4-triazolyl)methyl)amine (TBTA, 40 mM in DMSO, 1.6 mL), CuSO_4_ (20 mM in water, 1.6 mL), and sodium ascorbate (100 mM in water, 0.8 mL) was stirred at 37 ºC under an argon atmosphere for 1 hour. The resulting crude products were purified by preparative RP-HPLC to yield analytically pure compound **iRGD-MMAF** (16.0 mg, 77% yield). Purity was confirmed by LC-MS, and the product was obtained as a white powder. MS (ESI) calculated for C_117_H_187_N_29_O_35_S_2_ [M+2H]^2+^: 1311.16; found: 1311.7.

Synthesis of Alkyne-PEG3-Lys(FAM)-Glu-Val-Cit-PABC-MMAF (S13). Reagents and conditions: (a) bis(4-nitrophenyl) carbonate, DMAP, DMF, room temp, 2 hours; (b) MMAF, DIPEA, HOAt, DMF, 37 °C, overnight.; (c) 50% TFA/DCM, room temp, 1 hour; (d) FAM-NHS, DIPEA, DMF, room temp, overnight.

**Synthesis of Alkine-PEG3-Lys(Boc)-Glu(O*t*-Bu)-Val-Cit-PABC-OH (S9).**

Fmoc-Cit-PAB-O-resin was prepared according to the procedure described previously (Anami et al. Nat. Commun. 9:2512 (2018).). To remove a Fmoc-protecting group after each coupling, the resin was treated with 20% piperidine/DMF (5 mL) for 20 min and washed with DMF (5×3 mL) and DCM (5×3 mL). Propargyl-PEG3-acid (Broad Pharm, 3 equiv.), Fmoc-Lys(Boc)-OH (3 equiv.), Fmoc-Glu(O*t*-Bu)-OH (3 equiv.), or Fmoc-Val-OH (3 equiv.) was preactivated using HATU (3 equiv.) and DIPEA (4.5 equiv.) in DMF for 5 min, and the cocktail was added to the resin. The resin was agitated at room temperature for 1.5 hours. Completion of the coupling was verified by the Kaiser test. After each coupling step, the coupling cocktail was drained, and the resin was washed with DMF (5×3 mL) and DCM (5×3 mL). The resulting protected peptide resin was dried in vacuo and treated with a cocktail of 1% TFA/DCM at room temperature for 1 hour. The solution was concentrated in vacuo and the crude peptide was precipitated with cold diethyl ether (5–6 mL) followed by centrifugation at 2,000 × g for 3 minutes. The precipitate was washed by cold diethyl ether and centrifugation at 2,000 g for 5 minutes, three times. The resulting crude products were dried in vacuo and then used immediately in the next step without purification.

**Synthesis of Alkyne-PEG3-Lys(Boc)-Glu(OtBu)-Val-Cit-PABC-PNP (S10).**

Bis(2,4-dinitrophenyl) carbonate (81.4 mg, 0.265 mmol) and DMAP (13.1 mg, 0.107 mmol) were added to a solution of the crude peptide **S9** (53.1 mg, 0.054 mmol) in DMF (1 mL), and the mixture was stirred at room temperature for 2 hours under Ar. The reaction was quenched with 1% HCl/ACN (1 mL) at 0 ºC, then the crude products were purified by preparative RP-HPLC to afford analytically pure peptide **S10** (39.7 mg, 64.2% for overall yield, calculated based on the resin loading rate). Purity was confirmed by LC-MS. Off-white powder. MS (ESI) Calcd. For C_55_H_82_N_9_O_18_ [M+H]^+^: 1156.58. Found: 1156.5.

**Synthesis of Alkyne-PEG3-Lys(Boc)-Glu(OtBu)-Val-Cit-PABC-MMAF (S11).**

A solution of **S10** (39.7 mg, 34.4 µmol) in DMF (1 mL) was mixed with monomethyl auristatin F TFA salt (43.6 mg, 51.5 µmol), HOAt (4.7 mg, 34.4 µmol), and DIPEA (29.9 µL, 171.8 µmol). The resulting mixture was stirred overnight at 37 °C under argon atmosphere. The crude products were dried and DIPEA was removed in vacuo. The crude products were purified by preparative RP-HPLC to afford analytically pure peptide **S11** (25.0 mg, 42%). Purity was confirmed by LC-MS. Off-white powder. MS (ESI) Calcd. For C_88_H_142_N_13_O_23_ [M+H]^+^: 1749.03. Found: 1748.9.

**Synthesis of Alkyne-PEG3-Lys-Glu-Val-Cit-PABC-MMAF (S12).**

TFA (300 µL) and DCM (300 µL) were mixed and added to the dried compound **S11** at 0 ºC. After being stirred at room temperature for 1 hour, the reaction mixture was concentrated in vacuo and the crude peptide was precipitated with cold diethyl ether (18­­–20 mL) followed by centrifugation at 2,000 × g for 3 min (3 times). The resulting crude products were dried in vacuo and then used immediately in the next step without purification.

**Synthesis of Alkyne-PEG3-Lys(FAM)-Glu-Val-Cit-PABC-MMAF (S13).**

A solution of the crude compound **S12** (22.8 mg, 14.3 µmol) in DMF (1 mL) was mixed with 5(6)-carboxyfluorescein succinimidyl ester (FAM-NHS, 10.1 mg, 21.5 µmol), and DIPEA (7.4 µL, 42.9 µmol). The resulting mixture was stirred overnight at room temperature. The crude products were purified by preparative RP-HPLC to afford analytically pure peptide **S13** (11.1 mg, 40% for two steps). Purity was confirmed by LC-MS. Yellow powder. MS (ESI) Calcd. For C_100_H_136_N_13_O_27_ [M+H]^+^: 1950.97. Found: 1951.0.

Synthesis of FAM-iRGD-MMAF. Reagents and conditions: (a) phosphate buffer (pH 7), TBTA, CuSO_4_, ascorbic acid, DMSO, 37 ºC, argon atmosphere, 1 hour.

**Synthesis of FAM-iRGD-MMAF.**

The reaction mixture of **S13** (10 mM in DMSO, 0.23 mL), **S3** (10 mM in DMSO, 0.46 mL), 1 M phosphate buffer (pH 7, 0.69 mL), TBTA (40 mM in DMSO, 0.46 mL), CuSO_4_ (20 mM in water, 0.46 mL), and sodium ascorbate (100 mM in water, 0.23 mL) was stirred at 37 ºC under an argon atmosphere for 1 hour. The resulting crude products were purified by preparative RP-HPLC to yield analytically pure **FAM-iRGD-MMAF** (3.7 mg, 53% yield). Purity was confirmed by LC-MS, and the product was obtained as a white powder. MS (ESI) calculated for C_141_H_204_N_30_O_41_S_2_ [M+2H]^2+^: 1518.71; found: 1519.2.

**Azido-iRGD (S3)**


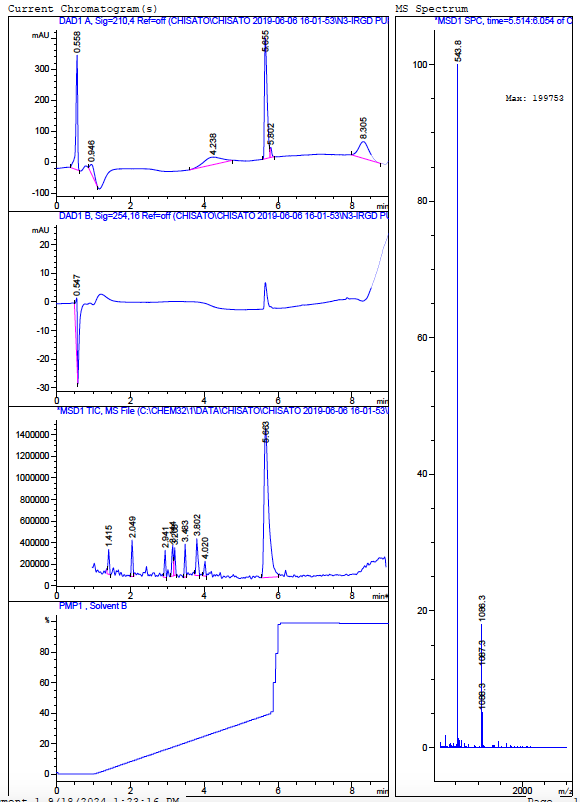


**Alkyne-PEG3-Glu(O*t*-Bu)-Val-Cit-PABC-PNP (S6)**

**
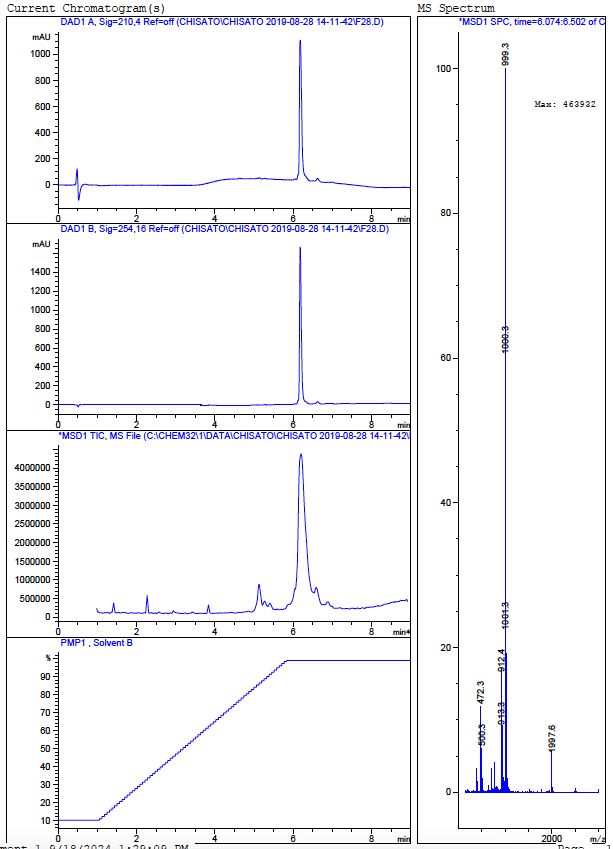
**

**Alkyne-PEG3-Glu-Val-Cit-PABC-MMAF (S8)**


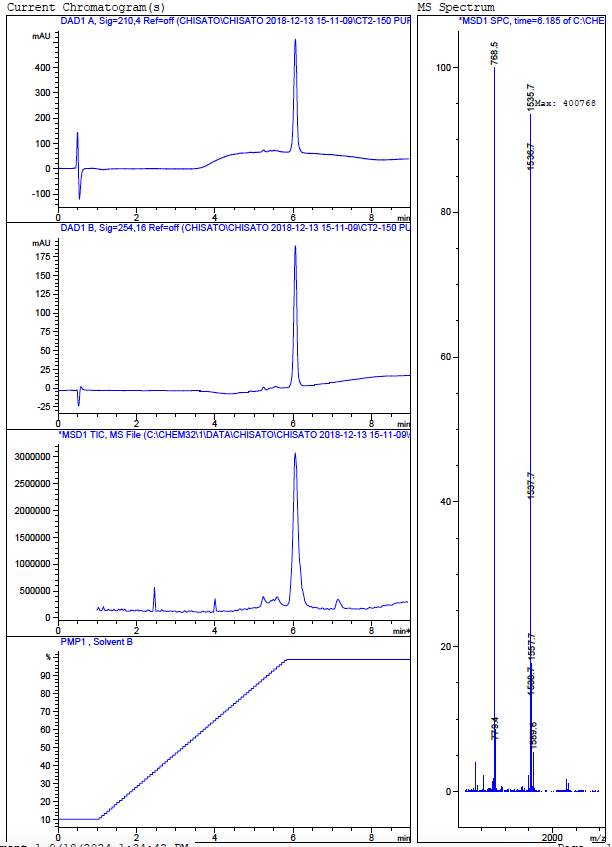


**iRGD-MMAF**

**
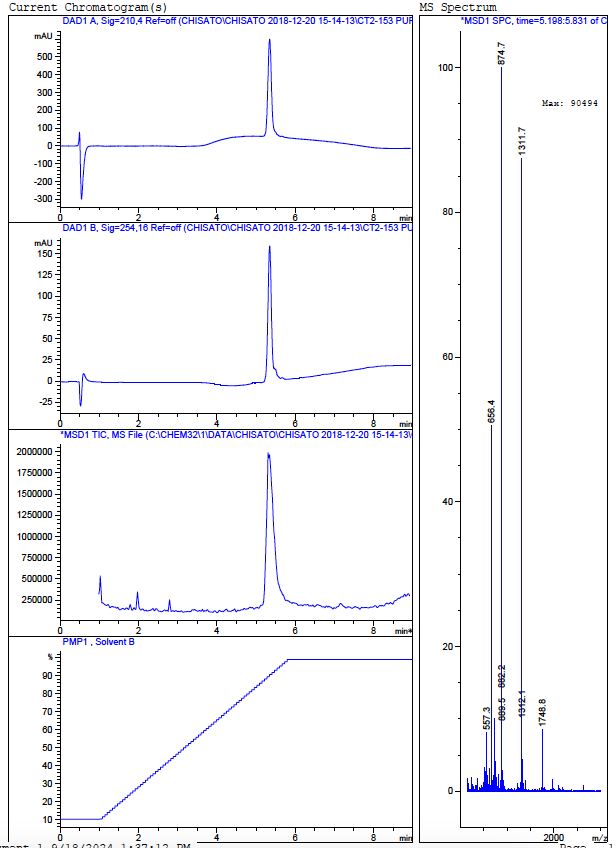
**

**Alkyne-PEG3-Lys(Boc)-Glu(O*t*-Bu)-Val-Cit-PABC-PNP (S10)**

**
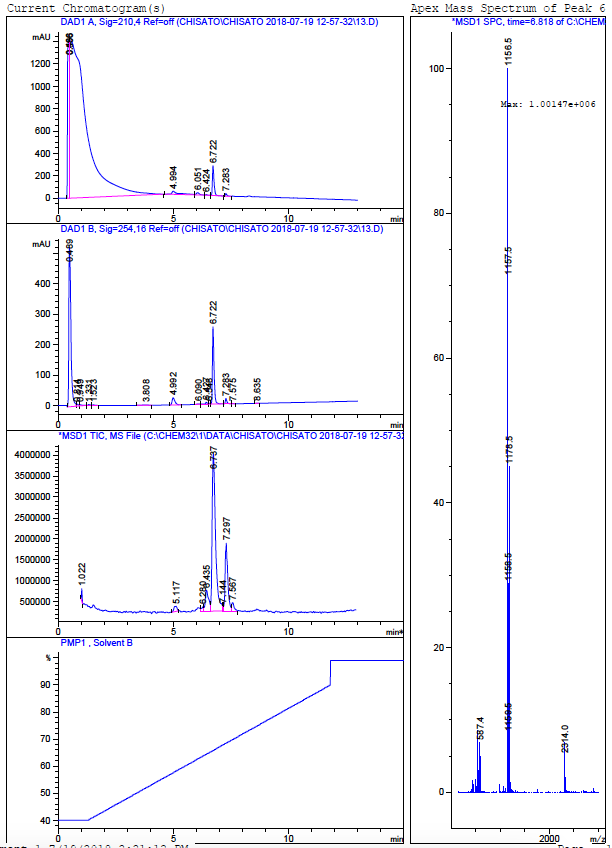
**

**Alkyne-PEG3- Lys(Boc)-Glu(O*t*-Bu)-Val-Cit-PABC-MMAF (S11)**


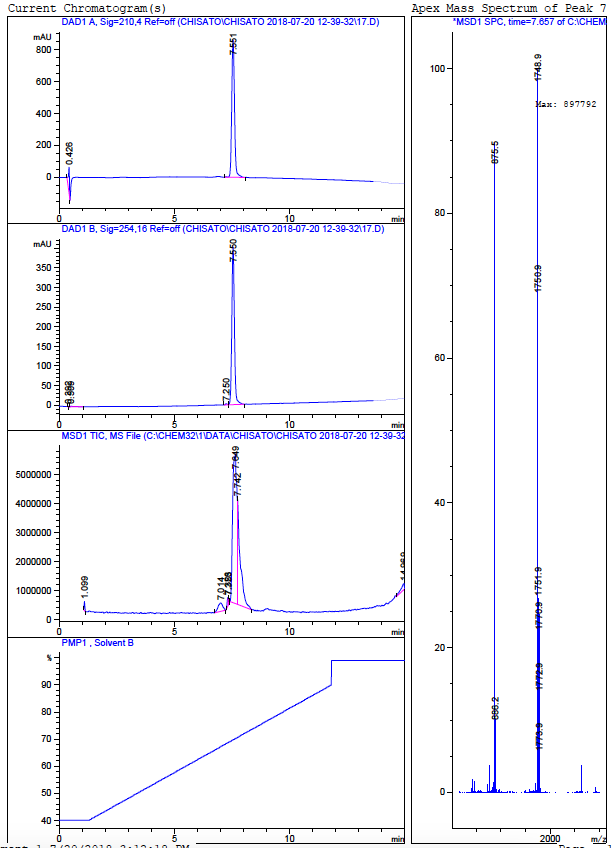


**Alkyne-PEG3- Lys(FAM)-Glu-Val-Cit-PABC-MMAF (S13)**

**
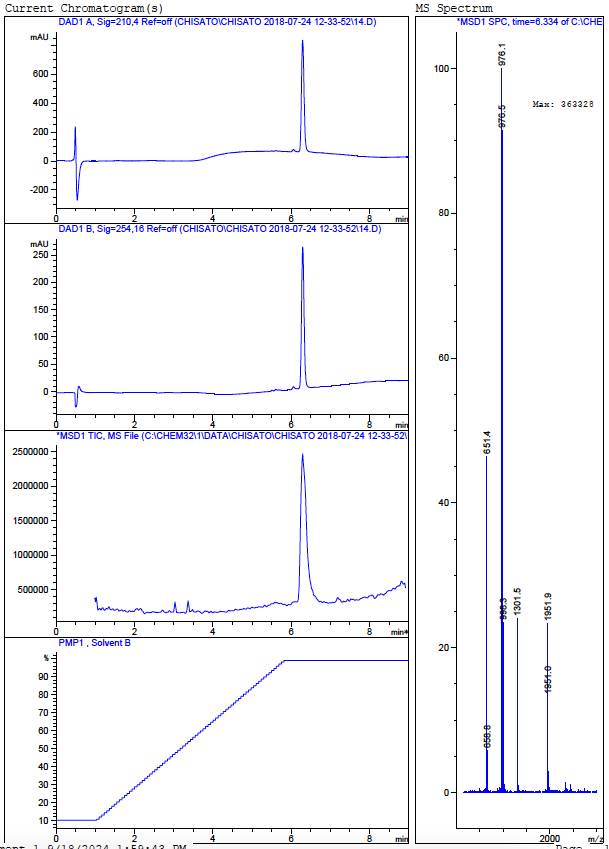
**

**FAM-iRGD-MMAF**


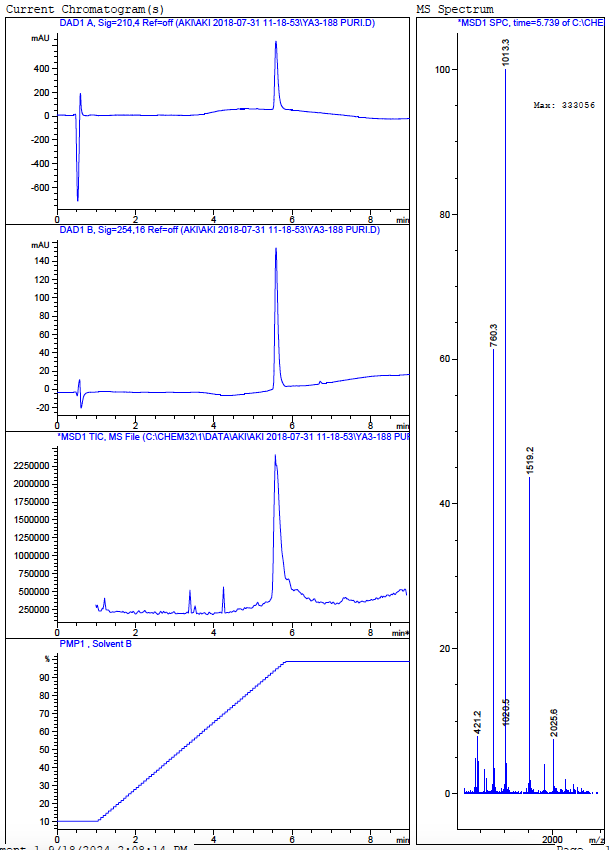

Supplement: S1 Information — (DOCX) [file pone.0331564.s001.docx]
