# Supplementary material for: A cytotoxic peptide-drug conjugate for tumor-specific delivery of co-injected molecules
Source: PLoS One. 2025 Sep 2;20(9):e0331564. doi: 10.1371/journal.pone.0331564 (PMC12404482; doi:10.1371/journal.pone.0331564)
Supplement: S1 Table — (DOCX) [file pone.0331564.s002.docx]

**Tumor size in mice with KRAS-Ink tumors following systemic treatment with MMAF or iRGD-MMAF**

|  |  | Day | | | | |
| --- | --- | --- | --- | --- | --- | --- |
| Treatment | Mouse No. | 0 | 2 | 4 | 7 | 9 |
| No injection | 1 | 96.8 | 126.0 | 194.7 | 446.9 | 564.0 |
|  | 2 | 114.5 | 137.3 | 224.6 | 424.1 | 589.9 |
|  | 3 | 209.5 | 309.4 | 512.5 | 736.5 | 884.3 |
| MMAF | 1 | 141.4 | 210.9 | 353.0 | 672.5 | 1022.9 |
|  | 2 | 189.2 | 171.5 | 377.7 | 488.5 | 533.6 |
|  | 3 | 97.5 | 288.0 | 255.9 | 351.8 | 644.0 |
|  | 4 | 163.4 | 171.5 | 331.7 | 484.7 | 967.0 |
| iRGD-MMAF | 1 | 173.8 | 169.0 | 288.6 | 429.7 | 494.7 |
|  | 2 | 124.5 | 288.0 | 242.1 | 412.1 | 470.9 |
|  | 3 | 181.8 | 98.3 | 340.4 | 472.1 | 558.1 |

Dimension: mm^3^
